# Lean adipose tissue macrophage derived exosome confers immunoregulation to improve wound healing in diabetes

**DOI:** 10.1186/s12951-023-01869-4

**Published:** 2023-04-12

**Authors:** Wenzheng Xia, Yunhan Liu, Xingyu Jiang, Minxiong Li, Shengwu zheng, Zewei Zhang, Xin Huang, Shenying Luo, Yimin Khoong, Meng Hou, Tao Zan

**Affiliations:** 1 Department of Plastic and Reconstructive Surgery, Shanghai Ninth People’s Hospital, Shanghai Jiao Tong University School of Medicine, Shanghai, 200011 China; 2 School of Chinese Materia Medica, Nanjing University of Chinese Medicine, Nanjing, China; 3 Shanghai Institute of Materia Medica, Chinese Academy of Sciences, Shanghai, China; 4 Department of Burn and Plastic Surgery, Shengli Clinical Medical College of Fujian Medical University, Fujian Provincial Hospital, Fuzhou, China; 5 Department of Oncology, Shanghai Pulmonary Hospital, Tongji University School of Medicine, Shanghai, 200433 China

**Keywords:** Diabetic wound healing, Lean adipose tissue macrophages, Exosomes, Macrophage polarization, Mir 222-3p/Bim signaling pathway

## Abstract

Chronic non-healing wounds, a prevalent complication of diabetes, are associated with increased mortality in diabetic patients. Excessive accumulation of M1 macrophages in diabetic wounds promotes inflammation and results in dysregulated tissue repair. Adipose tissue macrophages (ATMs) derived from healthy lean donors have the ability to improve glucose tolerance and insulin sensitivity, as well as modulate inflammation. MicroRNAs (miRs), which can be packaged into exosomes (Exos) and secreted from cells, serve as essential regulators of macrophage polarization. Here, we revealed that ATMs isolated from lean mice secrete miRs-containing Exos, which modulate macrophage polarization and promote rapid diabetic wound healing when administered to diabetes-prone db/db mice. The miRs sequence of tissue samples from wounds treated with Exos secreted by lean ATMs (Exos^Lean^) revealed that miR-222-3p was up-regulated. Further analyses showed that inhibiting miR-222-3p using a miR inhibitor impaired the macrophage-reprogramming effect of Exos^Lean^. In the excisional skin wound mouse model, locally inhibiting miR-222-3p disrupted healing dynamics and failed to modulate macrophage polarization. Mechanistic studies revealed a connection between miR-222-3p, Bcl2l11/Bim, an inflammatory response effector, macrophage polarization, and diabetic wound healing. In summary, Exos^Lean^ act as positive regulators of macrophage polarization by regulating miR levels in wounds and accelerating wound healing, and thus have important implications for wound management in diabetes.

**Graphic Abstract:**

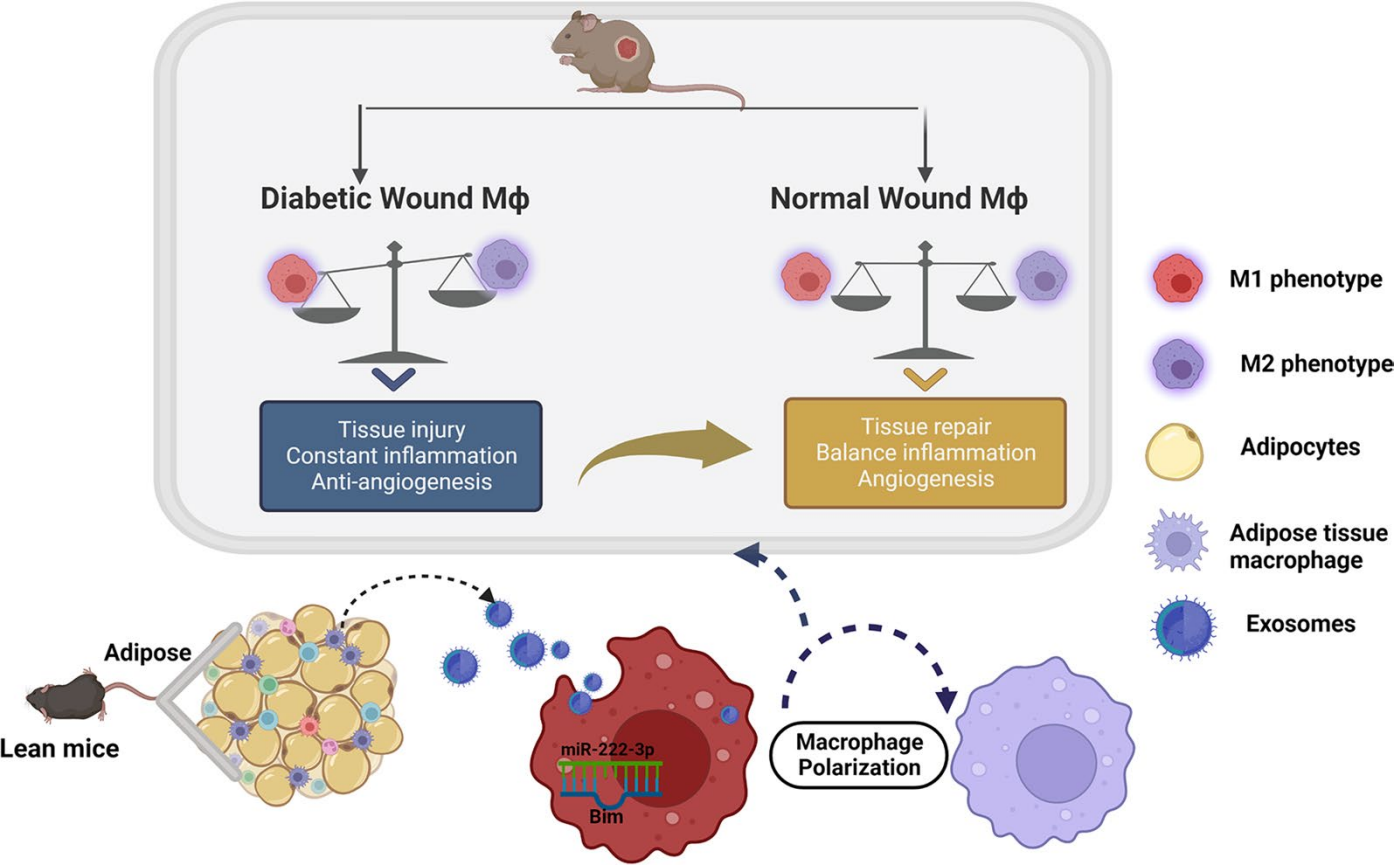

**Supplementary Information:**

The online version contains supplementary material available at 10.1186/s12951-023-01869-4.

## Introduction

Diabetes, one of five priority noncommunicable diseases (NCDs) [1], involve complex, long-term, and costly endeavors due to the chronic complications [2]. Type 2 diabetes mellitus (T2DM) leads to a number of long-term complications, and chronic diabetic wounds are a leading cause of hospitalizations for diabetics and a major morbidity associated with diabetes [3]. Chronic hyperglycemia and dyslipidemia are the hallmarks of T2DM [4]. Moreover, the chronic inflammatory state induced by hyperglycemia and dyslipidemia contributes to the occurrence of chronic diabetic wounds [5]. There are four phases in the wound healing process: hemostasis, inflammation, proliferation/repair, and remodeling. Macrophages are the most important immunomodulatory cells involved in wound healing [6]. Macrophages remove phagocytic debris and stimulate tissue granulation to help maintain tissue homeostasis during the healing process [7]. In T2DM patients, excessive and prolonged inflammation occurs at the wound site due to the predominance of M1 macrophages [8]. Consequently, the accumulation of M1 macrophages, which creates a harmful microenvironment, continuously promotes proteolysis and cell damage [9]. Thereafter, modulation of macrophage polarization may contribute to the healing of diabetic wounds.

The dysfunction of adipose tissue, characterized by metabolic disturbances, plays a critical role in T2DM pathogenesis [10]. In the context of obesity, adipose tissue becomes dysfunctional, loses its beneficial endocrine functions, and disrupts immune cell function, thus leading to tissue inflammation and fibrosis [11]. In the tissues of obese individuals with chronic diabetic wounds, obesity results in the accumulation of pro-inflammatory macrophages, a hallmark of tissue inflammation. Pro-inflammatory macrophages are the main source of inflammatory mediators and recruit, accumulate, and activate more pro-inflammatory macrophages in wound tissue, thereby leading to chronic inflammation-related failure in wound healing [12]. Therefore, it is crucial to achieve a balance between pro-inflammatory and anti-inflammatory macrophages in diabetic wound healing tissues. ATMs derived from healthy lean donors may be excellent candidates for achieving this balance due to their ability to improve glucose tolerance and insulin sensitivity, as well as modulate inflammation [13]. Emerging evidence suggests that in addition to cytokines, Exos, which are implicated in crosstalk between ATMs and metabolic organs, play critical roles in modulating metabolic and inflammatory homeostasis, which are important for diabetic wound healing [14, 15]. Previous studies have demonstrated that ATMs from lean mice contribute to tissue homeostasis by being polarized toward the M2 phenotype; however, obesity leads to an increase in the number of pro-inflammatory M1 ATMs, resulting in metabolic disorders associated with obesity [16]. In addition, to providing protection against obesity-induced insulin resistance, Exos^Lean^ also exerts similar protective effects against cell apoptosis [13, 17]. Moreover, Exos isolated from relatively lean donor adipose tissue drives the polarization of macrophages toward the M2 phenotype, resulting in a relative reduction in inflammation [18]. Thus, it is of great interest to investigate the ability of Exos^Lean^ to treat chronic diabetic wounds by restoring macrophage phenotype balance.

MiRs are regulatory noncoding RNAs primarily responsible for cell–cell interactions through Exos [19]. Exos containing numerous miRs can modulate the function of recipient cells locally or by entering the bloodstream and acting at distant sites [20]. A growing body of evidence suggests that exosomal miRs secreted by ATMs influence metabolic regulation [13, 14]. In addition, miRs expression in adipose tissue differs between obese and lean donors, and the levels of different miRs correlate with BMI to varying degrees [21, 22]. Evidence also suggests that Exos that are derived from ATMs and taken up by neighboring cells modulate the function of recipient cells, resulting in alleviation of insulin resistance, inflammatory homeostasis, and tissue protection [13]. This led us to hypothesize that ATMs from lean donors secrete exosomal miRs that can improve the healing of chronic diabetic wounds.

Here, we demonstrate that ATMs from lean animals secrete Exos containing miRs that are phagocytized by macrophages, leading to rebalancing of macrophage polarization and contributing to the therapeutic effect in chronic diabetic wounds. In addition, we hypothesize that miR-222-3p is the candidates treatment effector through a mechanism that is most likely associated with direct suppression of its target gene Bcl-2-like protein 11 (Bcl2l11)/Bim.

## Materials and methods

### Animals

All animal procedures associated with this work are authorized by the institution’s ethical committees. B6.BKS(D)-Leprdb/J (db/db) mice were applied as diabetes models. Low-fat diet fed mice were used as a lean model, as previously reported [23]. All mice were housed individually and had ad libitum access to food and water. Experimenters were not blinded to group assignment and outcome assessment.

### Wound healing model and treatment

The mice were anesthetized with 2.5% of isoflurane. The hair on the back of the mice was shaved off with an electric razor and sterilized using 70% alcohol. 8 mm skin wounds were made using a biopsy punch. From day 1 to day 5, 100 μg per 100 μL of Exos^Lean^ was injected at 4 injection sites (25 μL per site) around the wounds. The diameter of each wound (day 0, 5, 10, and 15) was measured using Photoshop to calculate the percentage of wound closure.

To induce local inhibition of expression of miR-222-3p, 1 nmol miR-222-3p inhibitor or negative control (ThermoFisher Scientific) was injected intradermally into the wound edges of mice in a total volume of 100 μL for each wound, as per manufacturer's instruction, as previously reported [24].

### Histological analysis and immunofluorescence

Tissue samples were fixed in 4% paraformaldehyde and 3 μm sections were cut from paraffin-embedded blocks. The sections were stained with standard Masson’s trichrome. Immunohistochemical staining for the presence of TNF-α was conducted in consecutive sections.

Immunofluorescent staining was performed with paraffin-embedded tissues and 4% PFA fixed macrophages. Following incubation with primary antibody, the slides were washed with 1X PBS and incubated for 1 h at room temperature with the secondary antibody. Sections were counterstained with DAPI mounting medium and analyzed. Fluorescence was detected under confocal laser scanning microscopy.

### Adipose tissue macrophage (ATMs) isolation

ATMs were isolated from mouse visceral adipose tissues (VATs). The preparation and sorting of ATMs by flow cytometry were performed as described previously [13]. Briefly, mechanically minced VATs were digested with collagenase II (Sigma-Aldrich) for 15 min at 37 °C. The collected digests were filtered with 100 μm cell strainers and centrifuged at 1000×*g* for 10 min. Pellets were incubated in RBC lysis buffer. For flow cytometry cell sorting, single-cell suspensions were incubated with fluorescence-tagged CD11b or F4/80 antibodies. CD11b+/F4/80+ macrophages were purified using FACS.

### Isolation and characterization of exosomes

Centrifuge the supernatant for 30 min (3000×*g*) to remove the precipitate, then add the supernatant into the ultrafiltration *M* tube and centrifuge for 30 min (3000×*g*) to obtain the upper chamber liquid. Exosome quick extraction solution was added to the filtered solution in a 1:5 ratio and stored at 4 °C for at least 12 h. Exosomes can be precipitated by centrifuging 10,000×*g* for 1 h. The isolation process was performed at 4 °C, and the exosomes were resuspended in PBS and stored at − 80 °C.

The morphology of exosomes was observed using transmission electron microscopy (TEM, JEM-1400plus). The size was determined through nanoparticle tracking analysis (NTA). Western blotting was used to detect the exosomal markers HSP70, TSG101, and CD63.

### MiRs sequencing

RNA was isolated using TRIzol reagent, and its concentration was quantified using a NanoDrop spectrophotometer while its integrity was evaluated. 3 μg of total RNA per sample was used as input material for the small RNA library. Following transforming the single-stranded DNA adaptor into a double-stranded DNA molecule, PCR amplification was performed. Finally, library’s quality was assessed using the Agilent Bioanalyzer 2100 system and DNA High Sensitivity Chips. DESeq2 was used to test for differential expression of small RNAs between samples.

### Quantitative real-time RT-PCR (qRT-PCR) assay

Trizol reagent (Invitrogen, USA) was used to extract total RNA from treated cells. Then, DNA reverse transcription was performed. qRT-PCR was carried out using SYBR Green Master (ROX) (Roche, USA). The mRNA levels were calculated relative to the control Gapdh (for mRNA) or U6 (for miRs) using the 2^−ΔΔCq^ method, as previously reported [25].

### Immunoblots

Cells were lysed using RIPA solution, and protein content was determined using a BCA assay. The electrophoretically separated protein was transferred to PVDF membranes. After blocking, the membranes were separately incubated overnight with primary antibodies, followed by HRP-conjugated secondary antibodies. The stained protein bands were visualized using a Bio-Rad ChemiDoc XRS imaging system and analyzed using Quantity One software.

### Dual luciferase reporter assays

The luciferase reporter assay was performed to validate the 3′-UTR binding site of Bim with miR-222–3p, according to previous research [26]. Briefly, HEK293T cells were seeded in 96-well plates and co-transfected with target plasmids and mimic/miR control miR-222-3p. After 24 h of transfection, luciferase activity was determined and the reporter assay was performed according to the manufacturer's protocol (Promega). Renilla luciferase activity was normalized relative to activity of firefly luciferase and expressed as a percentage of the control.

### Flow cytometry

The expression of cell surface markers for M1 or M2 phenotypes was evaluated using flow cytometry, as described previously [27]. Briefly, following treatment, RAW 264.7 cells were surface-stained with antibodies specific for CD86 (M1 marker) and CD206 (M2 marker) for 30 min at 4 °C. After additional washing procedures, the cells were analyzed using a BD FACS AriaII flow cytometer and FlowJo software.

### Statistical analysis

The data was presented as mean ± standard deviation. The statistical significance of differences between groups was evaluated using one-way or repeated measures ANOVA. Two groups were compared using the student’s t-test or paired t-test. *P* < 0.05 was considered to be statistically significant.

## Results

### Dysregulated macrophage polarization and wound healing in diabetic mice

**Fig. 1:**
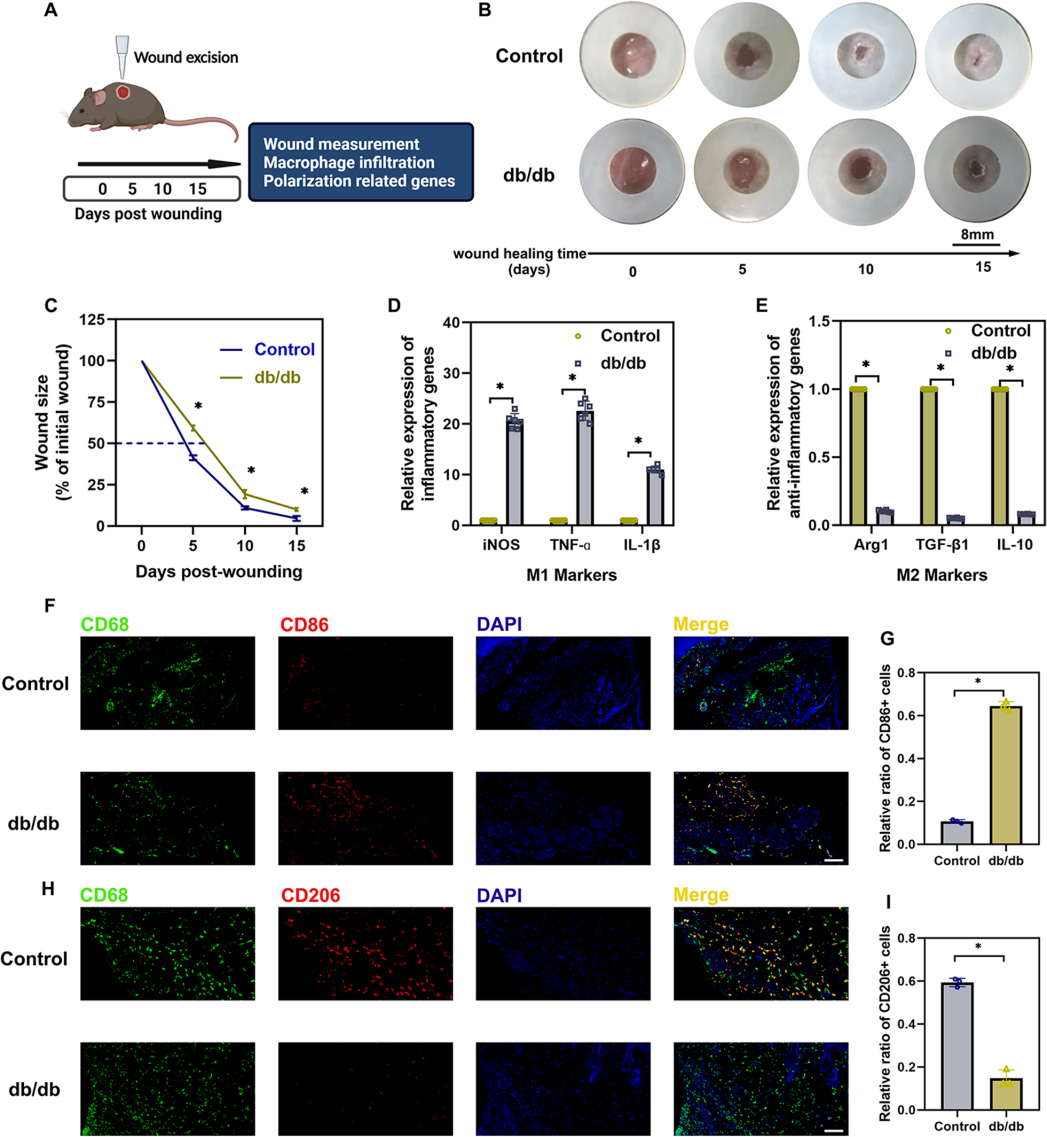
Dysregulated macrophage polarization and wound healing in diabetic mice. **A** Schematic illustration of wound healing and macrophage polarization. **B** Representative images of wound closure in control and db/db mice. **C** The relative wound area was calculated. **D**, **E** The expression levels of M1 markers and M2 markers. **F**, **G** Immunofluorescence staining images showing the localization of CD68+/CD86+ M1 macrophages (**F**) and statistical analysis of the proportion of M1 macrophages (**G**). **H**, **I** Immunofluorescence staining images showing the localization of CD68+/CD206+ M2 macrophages (H) and statistical analysis of the proportion of M2 macrophages (**I**). Scale bars: 100 μm; ^*^P < 0.05 by Student’s *t test*s (n = 3)

Chronic non-healing wounds impair the quality of life of diabetic patients [28]. Leptin receptor-deficient db/db mice were employed to assess the relevance of macrophage polarization to diabetic wound healing (Fig. 1A). As expected, the skin excision model showed that wound healing was impaired in the db/db mouse model, and a delay of nearly three days was observed before the wound reached half-maximal closure (Fig. 1B and C). To test whether dysregulated macrophage polarization contributed to the impaired diabetic wound healing, the expression of M1 and M2 markers at the mRNA level was tested using qRT‒PCR. The results showed a significant increase in the levels of M1 markers (iNOS, TNF-ɑ, and IL-1β) (Fig. 1D), while the levels of M2 markers (Arg1, TGF-β1, and IL-10) (Fig. 1E) decreased. In addition, immunofluorescence staining for CD86 (M1 marker) and CD206 (M2 marker) was performed. As shown in Fig. 1F–I, the proportion of M1-like macrophages (CD68 +, CD86 +) in the db/db groups was significantly higher than that in the control group (Fig. 1F and G), while the proportion of M2-like macrophages (CD68+, CD206+) was decreased (Fig. 1H and I).

### ATM-Exos derived from lean mice accelerate wound healing

**Fig. 2:**
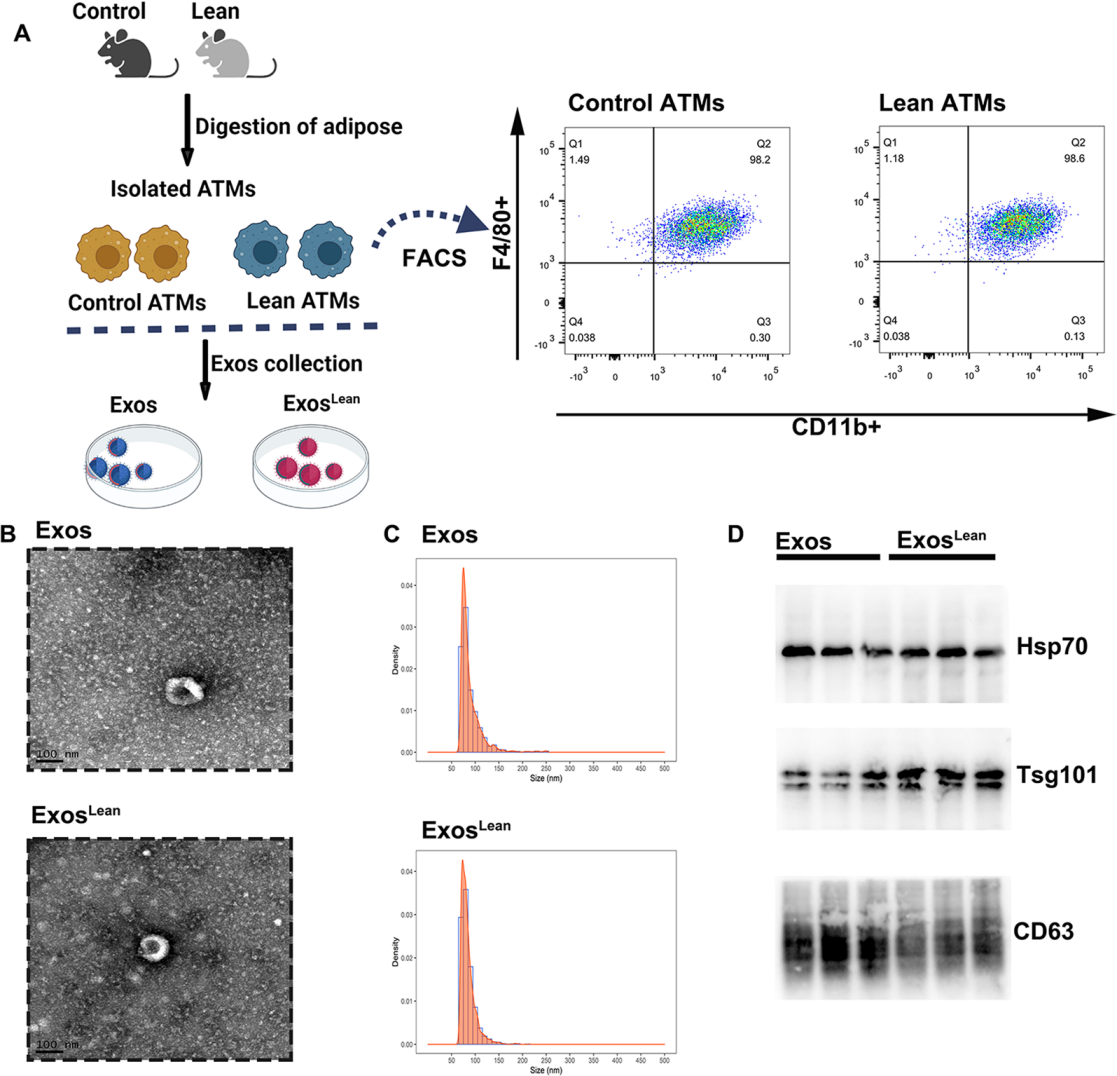
Exos isolation and characterization. **A** Schematic diagram of Exos isolated from ATMs collected from control mice and lean mice. Exosomal collection was confirmed using TEM, NTA and western blot analysis. **B** Representative TEM image. **C** Size range of Exos validated using NTA. **D** Representative western blot images showing that the exosomal markers HSP70, TSG101, and CD63 were expressed in Exos and Exos^Lean^

As it has been reported that ATM-secreted Exos derived from lean animals alleviate chronic inflammation and insulin resistance [13], we assessed whether Exos derived from lean animals hasten wound healing. Exos were isolated from lean donors and control mice (Fig. 2A), and electron microscopy (Fig. 2B) and NTA (Fig. 2C) revealed that the particles had a diameter of approximately 100 nm. The protein expression of the Exos markers HSP70, TSG101, and CD63 was measured in these fractions (Fig. 2D).

**Fig. 3:**
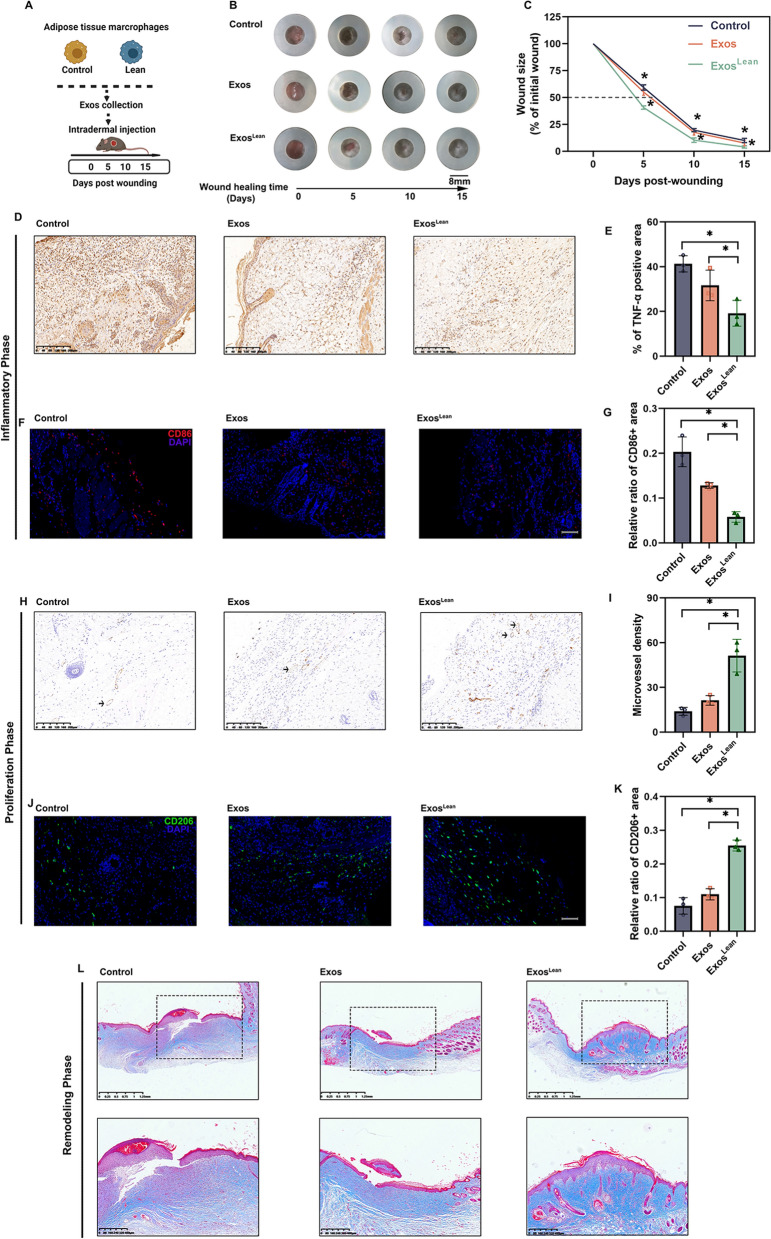
ATM-Exos derived from lean mice accelerate wound healing. **A** Schematic illustration of Exos treatment. **B** Representative images of wound closure in the different treatment groups. **C** The relative wound area was calculated. **D** Representative images of immunohistochemical staining for TNF-α. Scale bars: 200 μm. **E** Statistical analysis of TNF-α levels. **F** Immunofluorescence staining image showing the localization of CD86+ M1 macrophages. Scale bars: 100 μm. **G** Statistical analysis of the proportion of M1 macrophages. **H** Immunofluorescence staining for CD31. Scale bars: 200 μm. **I** Statistical analysis of CD31 levels. **J** Immunofluorescence staining images showing the localization of CD206+ M2 macrophages. Scale bars: 100 μm. **K** Statistical analysis of the proportion of M2 macrophages. **L** Representative images of Masson's trichrome staining. ^*^P < 0.05 by one-way analysis of variance (n = 3)

Next, we tested whether Exos^Lean^ affects the wound healing dynamics. Mice with full-thickness skin wounds were topically administered Exos^Lean^, Exos, and PBS as a control (Fig. 3A). While Exos^Lean^ accelerated wound healing, neither Exos nor PBS improved wound healing (Fig. 3B and C). Additionally, three stages of tissue repair were assessed. During the initial inflammation phase of wound healing, the level of inflammation was abnormally high in db/db mice, as evidenced by the elevated expression of TNF-α (Fig. 3D and E) and accumulation of M1 macrophage (Fig. 3F and G). TNF-α expression and M1 accumulation were decreased in Exos^Lean^-treated mice compared to db/db mice (Fig. 3D to G). In the proliferation phase, macrophage polarization and neovascularization are critical for angiogenesis and fibroblast infiltration [29]. Thus, immunostaining for platelet endothelial cell adhesion molecule-1 (CD31) (Fig. 3H and I and CD206 (Fig. 3J and K) was performed to assess the presence of newly generated vessels and M2 accumulation. The wound sections of Exos^Lean^-treated mice contained a greater number of CD31- and CD206-positive cells than those of the control and Exos-treated mice (Fig. 3H to K). During the remodeling phase, extracellular matrix (ECM) deposition and remodeling are critical for wound healing. Therefore, Masson trichrome staining was performed to evaluate ECM deposition. Wound tissues from Exos^Lean^-treated mice exhibited a relatively intact epidermal layer and appropriate ECM deposition (Fig. 3L). However, administration of control Exos alone did not result in such a significant improvement in wound healing.

### MiR-222-3p enhances wound healing

**Fig. 4:**
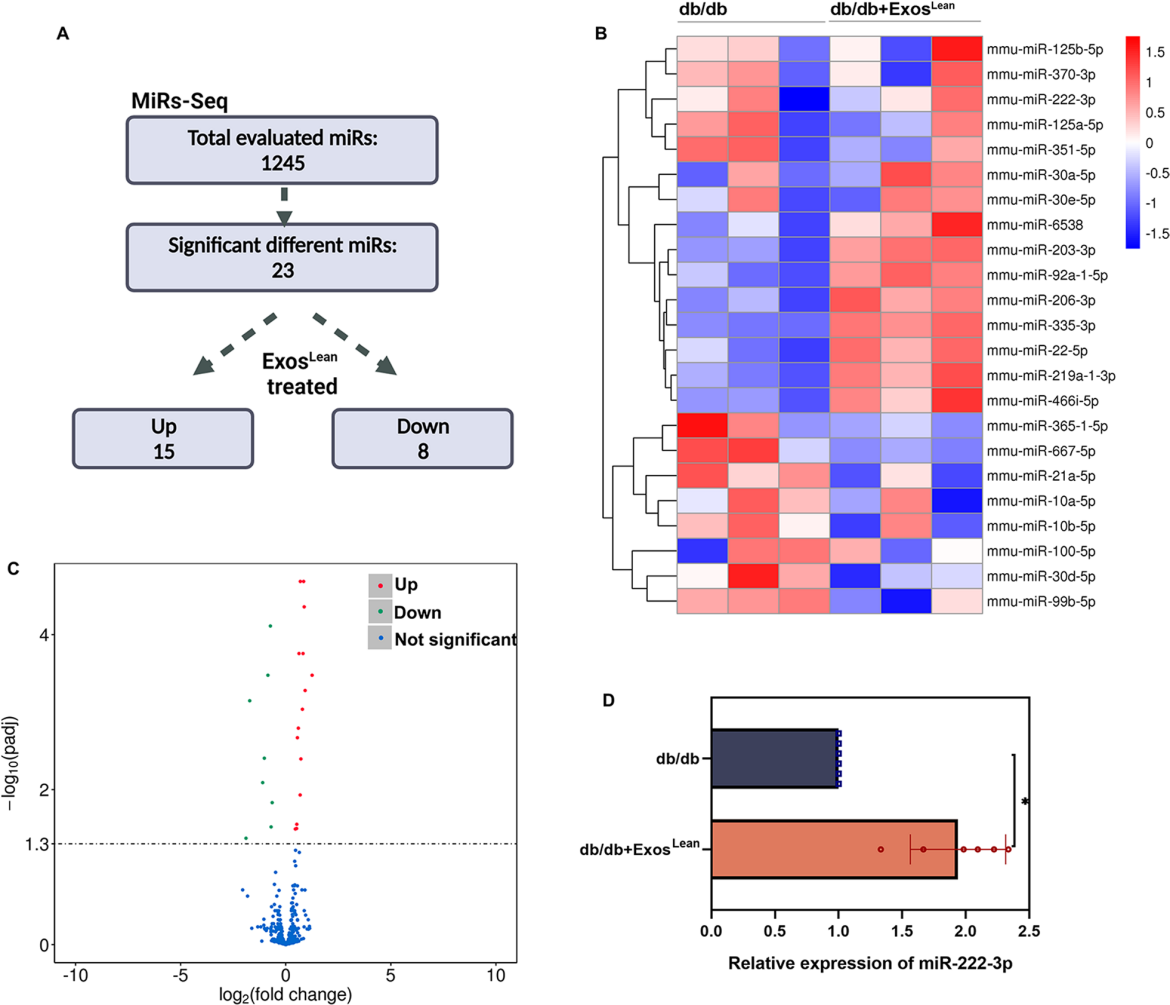
MiRs are differentially regulated by Exos^Lean^ treatment in db/db mice. **A** MiRs-Seq results. **B** Heatmap of differentially expressed miRs in the Exos^Lean^-treated and control groups. **C** A volcano plot was used to visualize the relationship between the fold change in expression and statistical significance. **D** MiR-222-3p expression was validated by qRT‒PCR. ^*^P < 0.05 by Student’s *t test* (n = 6)

**Fig. 5:**
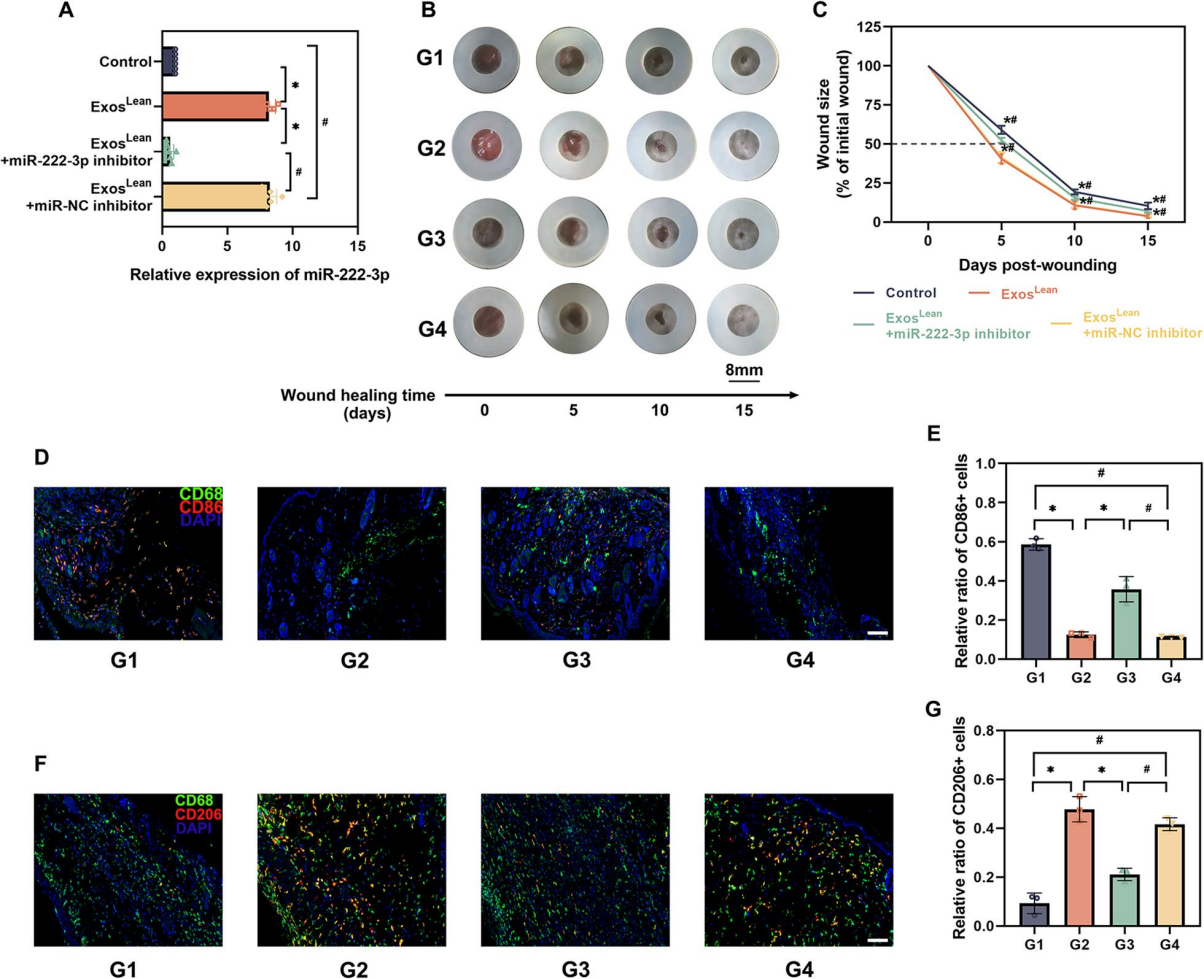
MiR-222-3p enhances wound healing. **A** QRT‒PCR analysis of miR-222-3p expression in the wound edges after miR-222-3p inhibitor or miR-NC inhibitor injection. ^*^P < 0.05 by one-way analysis of variance (n = 6). **B** Representative images of wound closure in the different treatment groups. **C** The relative wound area was calculated. **D**, **E** Immunofluorescence staining image showing the localization of CD68+/CD86+ M1 macrophages and statistical analysis of the proportion of M1 macrophages. (F, G) Immunofluorescence staining image showing the localization of CD68+ /CD206 + M2 macrophages and statistical analysis of the proportion of M2 macrophages. Scale bars: 100 μm; G1: control; G2: Exos^Lean^; G3: Exos^Lean^ + miR-222-3p inhibitor; G4: Exos^Lean^ + miR-NC inhibitor; ^*^P < 0.05, ^#^*P* < 0.05 by one-way analysis of variance (n = 3)

To determine the mechanism underlying the therapeutic effect of Exos^Lean^ on diabetic wound healing, miRs sequencing analysis was performed on db/db mice and db/db mice treated with Exos^Lean^ (Fig. 4A and B). During the Exos^Lean^ induced diabetes wound healing process, an equivalent response of miRs was observed, with 15 miRs being significantly up-regulated and8 miRs being significantly down-regulated (Fig. 4C). MiR-222-3p expression was further validated using qRT‒PCR (Fig. 4D). MiR-222-3p was chosen because it modulates inflammation and may be involved in tissue regeneration [30]. Using a synthetic miR-222-3p inhibitor, we then examined whether inhibiting miR-222-3p expression influences the wound healing dynamics. Local injection of the synthetic miR-222-3p inhibitor decreased miR-222-3p expression five days post-wounding (Fig. 5A). Notably, Exos^Lean^ improved wound closure (Fig. 5B and C) and markedly decreased the inflammatory burden in wounds with fewer M1 cells (Fig. 5D and E) and more M2 cells (Fig. 5F and G). However, local miR-222-3p inhibition reversed this accelerated wound healing and inflammatory modulation, as indicated by disruption of wound healing (Fig. 5B and C) and impaired macrophage polarization (Fig. 5D to G).

### Regulation of exosomal miR-222-3p in reprogramming of M1 to M2

**Fig. 6:**
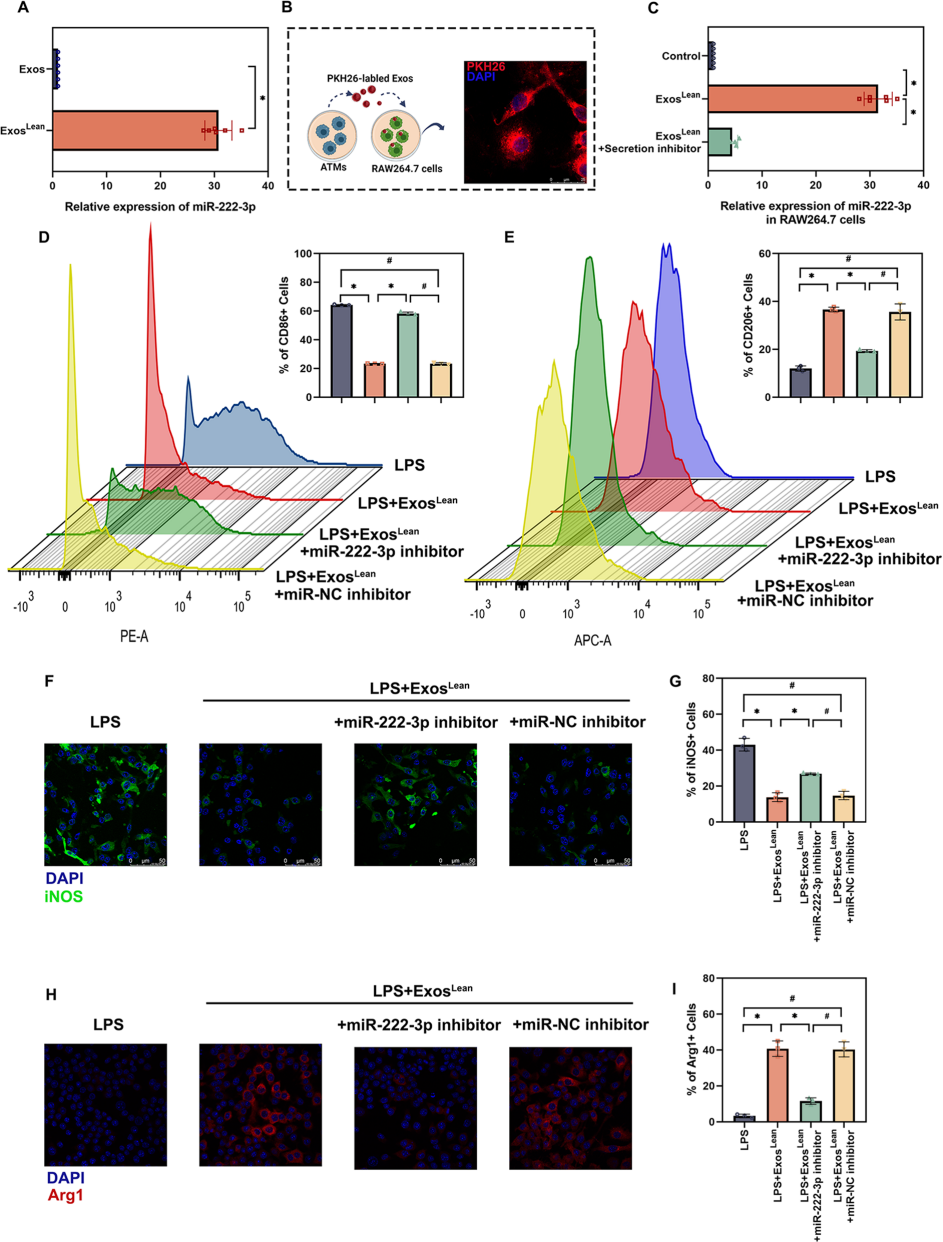
Regulation of exosomal miR-222-3p in reprogramming of M1 to M2. **A** MiR-222-3p expression in Exos was validated by qRT‒PCR. ^*^P < 0.05 by paired t test (n = 6). **B** Exos were labeled with PKH26 and then added to the culture medium of RAW264.7 macrophages. **C** The effects of an extracellular vesicle (EV) secretion inhibitor on Exos-dependent miR delivery. ^*^P < 0.05 by repeated-measures analysis of variance (n = 6). LPS-activated RAW 264.7 cells were treated with Exos^Lean^, Exos^Lean^ + miR-222-3p inhibitor, or Exos^Lean^ + miR-NC inhibitor. **D** Flow cytometry analysis and quantification of the relative abundance of CD86+ cells. **E** Flow cytometry analysis and quantification of the relative abundance of CD206+ cells. **F**, **G** Immunofluorescence staining image showing the localization of iNOS + cells and statistical analysis of the proportion of these cells. **H**, **I** Immunofluorescence staining image showing the localization of Arg1 + cells and statistical analysis of the proportion of these cells. Scale bars: 50 μm; ^*^P < 0.05, ^#^*P* < 0.05 by repeated-measures analysis of variance (n = 3)

Given that Exos^Lean^ induced inflammation balance during accelerated wound healing by modulating miR-222-3p, its function as an Exos regulator of inflammation was anticipated. We first determined whether miR-222-3p accumulated in Exos^Lean^. The qRT‒PCR results demonstrated that miR-222-3p was elevated in Exos^Lean^ relative to Exos (Fig. 6A). Next, we explored whether RAW264.7 macrophages can take up Exos^Lean^. PKH26-labeled Exos were added to the Raw264.7 cell culture medium. The presence of red fluorescence in macrophages indicated effective uptake of Exos^Lean^ (Fig. 6B). Furthermore, the secretion inhibitor GW4689 was applied to block Exos production and miR-222-3p delivery from ATMs to macrophages. This inhibitor decreased miR-222-3p expression in macrophages, indicating that miRs were predominantly transported in an Exos-dependent manner (Fig. 6C). Flow cytometry was utilized to quantitatively analyze the proportion of macrophages. LPS-treated control macrophages exhibited high levels of CD86(M1 polarization induction). In contrast, the M1 marker CD86 was downregulated in Exos^Lean^-treated macrophages, and this anti-inflammatory effect was reversed by miR-222-3p inhibition in macrophages (Fig. 6D). Exos^Lean^ consistently increased the proportion of CD206 + anti-inflammatory (M2-like) macrophages, whereas inhibition of miR-222-3p decreased the proportion of CD206 + macrophages (Fig. 6E). In addition, immunofluorescence staining for M1 macrophages (iNOS) and M2 macrophages (Arg1) was performed to investigate the effects of Exos^Lean^ on the transformation of M1 macrophages into M2 macrophages. The Exos^Lean^ group had a lower level of the M1 marker iNOS (Fig. 6F and G) and a higher level of the M2 marker Arg1 (Fig. 6H and I). Nevertheless, miR-222-3p inhibition appeared to inhibit transformation (Fig. 6F and I).

### Bim is a bona fide miR-222-3p target gene

**Fig. 7:**
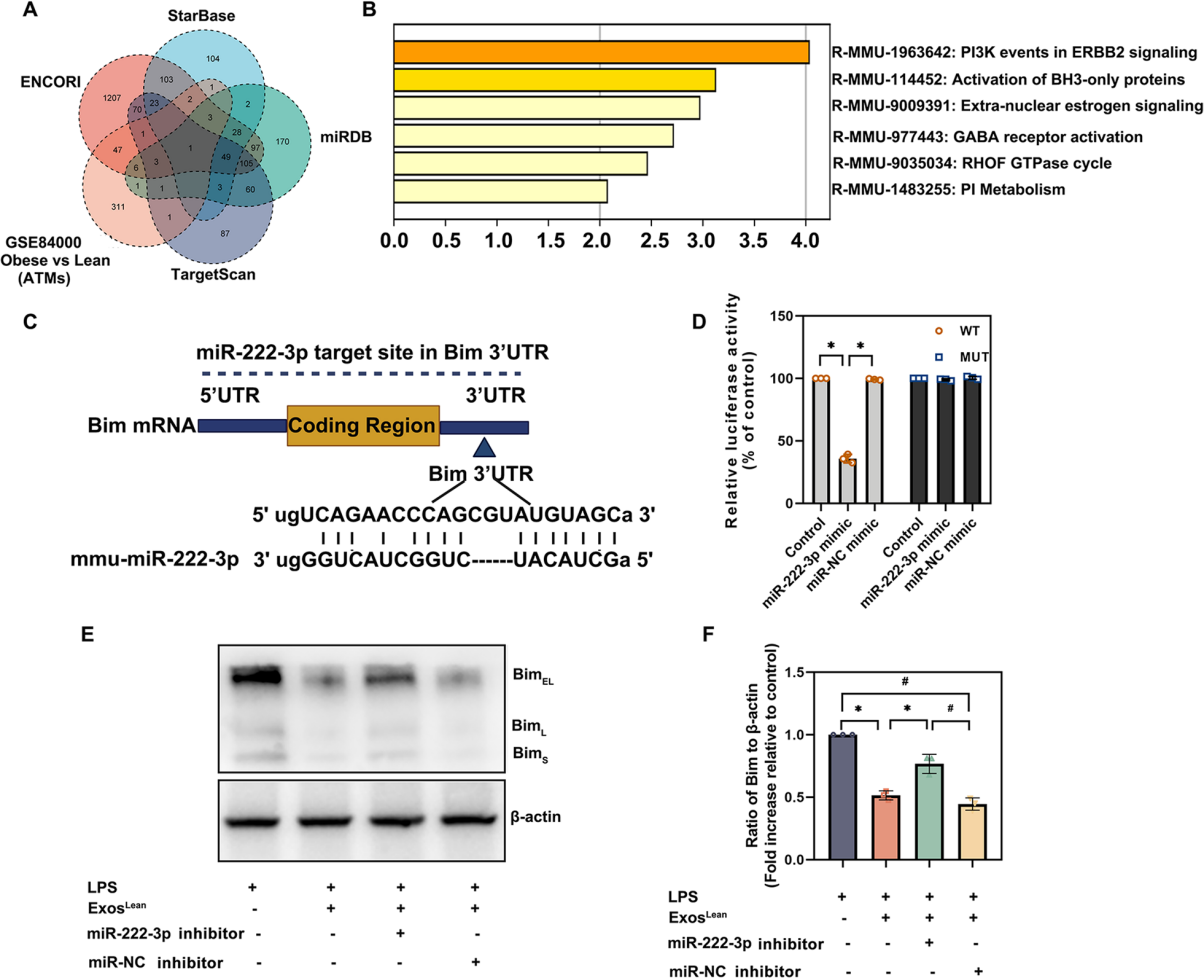
MiR-222-3p directly targets Bim. **A** Venn diagram showing the number of genes. significantly upregulated in ATMs from obese donors compared with ATMs from lean donors according to microarray analysis and the number of potential targets of miR-222-3p predicted by target prediction algorithms. **B** Enrichment analysis of pathways performed by Metascape. **C** Predicted binding sites between miR-222-3p and the Bim 3'-UTR. **D** Dual-luciferase assays were performed in HEK293T cells after co-transfection with Bim 3'-UTR wild-type (WT) or mutant (MUT) plasmids and miR-222-3p mimic or miR-NC mimic. ^*^P < 0.05 compared with the WT group by repeated-measures analysis of variance (n = 3). **E**, **F** Western blotting was used to analyze Bim and β-actin protein levels in RAW264.7 cells treated with LPS, LPS + Exos^Lean^, LPS + Exos^Lean^ + miR-222-3p inhibitor, or LPS + Exos^Lean^ + miR-NC inhibitor. ^*^P < 0.05, ^#^*P* < 0.05 by repeated-measures analysis of variance (n = 3)

To further explore the mechanisms by which exosomal miR-222-3p induces the transformation of M1 macrophages into M2 macrophage, the target gene of miR-222-3p was identified. A Venn diagram was used to represent the number of genes significantly upregulated in ATMs from obese donors compared to those from lean donors based on microarray analysis (GSE84000) [23] and the number of potential targets of miR-222-3p predicted by target prediction algorithms (Fig. 7A). Analysis of the biological processes and pathways associated with a subset of potential miR-222-3p targets by metascape (https://metascape.org/) revealed that “Activation of BH-3 only proteins” may play a role in miR-222-3p modulation (Fig. 7B). Bim was identified as a potential miR-222-3p target. The dual-luciferase gene reporter assay was used to confirm the presence of a potential binding site between miR-222-3p and Bim (Fig. 7C). Weaker relative luciferase activity was observed in the WT Bim + miR-222-3p mimic group (Fig. 7D). An increase in the Bim protein level in macrophages was confirmed using western blotting. Moreover, Exos^Lean^ significantly inhibited Bim expression in macrophages, whereas inhibition of miR-222-3p in macrophages restored the expression of Bim (Fig. 7E and F). We also investigated the effect of Exos^Lean^ on the expression of Bim in vivo. Bim expression decreased significantly in db/db mice treated with Exos^Lean^, whereas this inhibition was reversed by wound-locally transfected miR-222-3p inhibitor (Additional file 1: Fig. S1A and 1B).

**Fig. 8:**
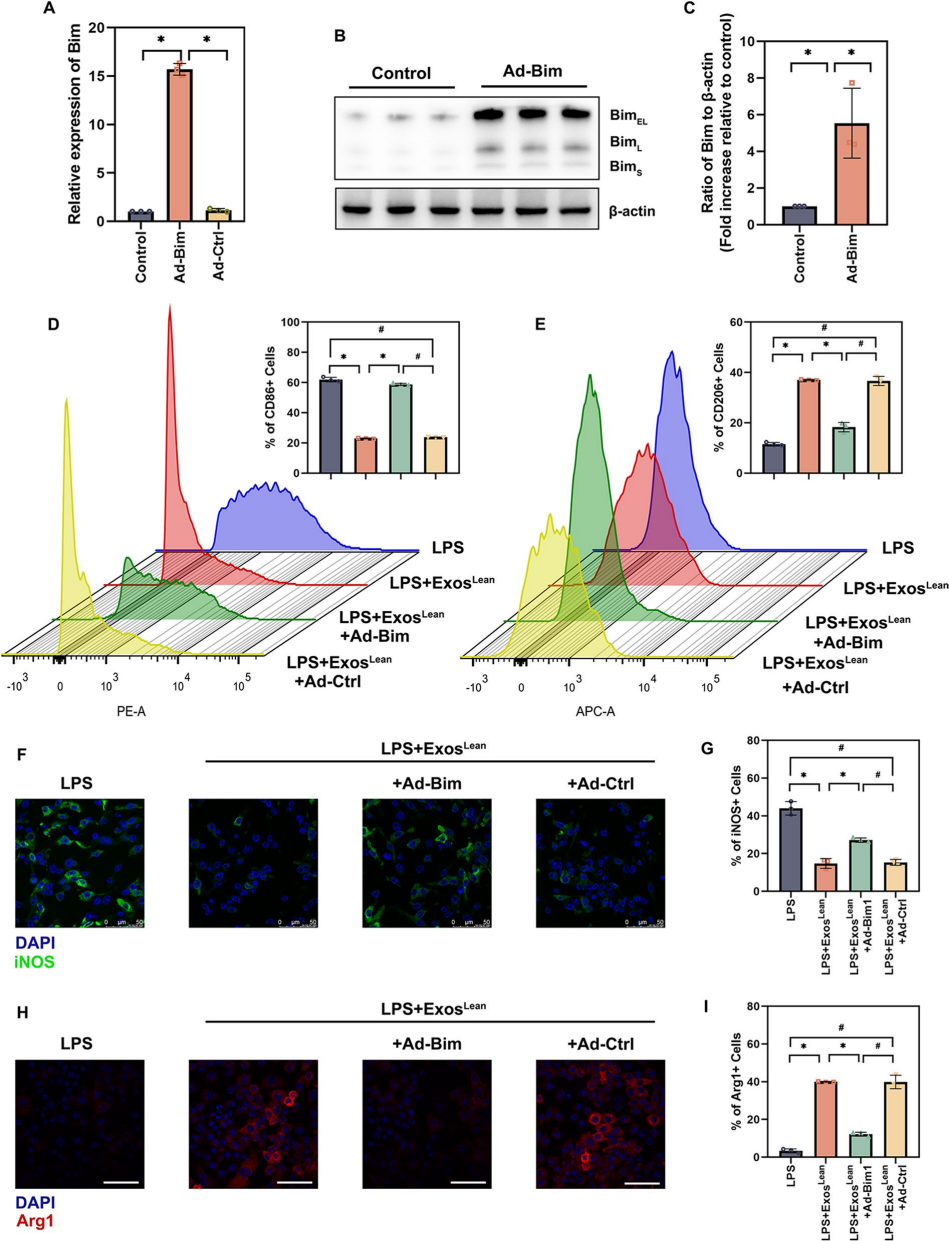
Overexpression of Bim inhibited macrophage polarization. **A** Bim mRNA expression was measured by qRT‒PCR. **B**, **C** Western blotting was used to analyze Bim and β-actin protein levels. ^*^P < 0.05 by repeated-measures analysis of variance (n = 3). **D** Flow cytometry analysis and quantification of the relative abundance of CD86+ cells. **E** Flow cytometry analysis and quantification of the relative abundance of CD206+ cells. **F**, **G** Immunofluorescence staining image showing the localization of iNOS + cells and statistical analysis of the proportion of these cells. (H, I) Immunofluorescence staining image showing the localization of Arg1 + cells and statistical analysis of the proportion of these cells. Scale bars: 50 μm; ^*^P < 0.05, ^#^*P* < 0.05 by repeated-measures analysis of variance (n = 3)

We next investigated whether the downregulation of Bim by exosomal miR-222-3p modulates macrophage polarization. Comparing cells infected with Ad-Bim to cells infected with Ad-Control (Ad-Ctrl), adenoviruses (Ads) coding Bim (Ad-Bim) were utilized to elevate Bim expression in macrophages. qRT‒PCR results indicated that Bim mRNA expression was significantly upregulated in cells infected with Ad-Bim (Fig. 8A). Western blotting was subsequently utilized to further confirm the effects of recombinant Ad vector-mediated gene overexpression. As shown in Fig. 8B and C, Ad-Bim significantly increased the expression of Bim. The increase in M1 macrophages (Fig. 8D) and decrease in M2 macrophages (Fig. 8E) indicated that upregulation of the expression of the miR-222-3p target gene Bim is sufficient to inhibit Exos^Lean^-mediated macrophage modulation and that Bim is an important regulator of this process. Similarly, immunofluorescence staining of M1 macrophages (iNOS) and M2 macrophages (Arg1) presented more iNOS-positive macrophages (Fig. 8F and G) and fewer Arg1-positive macrophages (Fig. 8H and I) in Bim-overexpressing macrophages compared to Exos^Lean^-treated macrophages. These results indicate that Bim is an exosomal miR-222-3p-sensitive macrophage polarization regulator.

As the continued inhibition of Bim by Exos^Lean^ is associated with macrophage polarization, we examined whether overexpression of Bim affects the pro-healing and immune-modulating effects induced by Exos^Lean^. Using AAV9-Bim vectors, Bim overexpression was induced in vivo. Wound healing was impaired in mice treated with Exos^Lean^ + AAV9-Bim (Additional file 2: Fig. S2A and 2B). In addition, locally transfection with Bim was accompanied with dysregulated macrophage polarization (Additional file 2: Fig. S2C and 2D).

## Discussion

The impairment of wound healing in individuals with T2DM is associated with substantial morbidity and mortality [31]. Therefore, considerable effort has been devoted to elucidating the pathogenesis of diabetic non-healing wounds and identifying novel treatments. The four phases of wound healing are primarily mediated by macrophages [9]. Here, we show that T2DM impairs wound healing through immune imbalance, disrupting the transformation of M1 macrophages into M2 macrophages, thereby interfering with all phases of the wound healing process. As an important component of antimicrobial defense during the inflammatory phase, M1 (pro-inflammatory) macrophages are the most important inflammatory cells in wound healing as they phagocytose pathogens [32]. However, diabetic wounds exhibit dysregulated and persistent M1 (pro-inflammatory) macrophage polarization, whereas normal wounds exhibit a transition to M2 (pro-healing) macrophages [33]. Recent research has shown that various epigenetic alterations evoked by a hyperglycemic environment led to elevated inflammatory cytokine expression, thus promoting M1 macrophage polarization [34]. Here, we demonstrated that there the expression of pro-inflammatory cytokines, such as TNF-a, a glucose intolerance factor that links immune cells to metabolic dysfunction, is increased in the wound edge. Moreover, failure of macrophage phenotype transformation inhibits tissue granulation stimulation during the proliferation phase. During the proliferation phase, macrophage phenotype transformation inhibits inflammation while promoting angiogenesis [35]; however, in our research, we observed an insufficient number of new vessels along with macrophage polarization defects. During the remodeling phase, which typically last weeks to years, fibroblasts, with the assistance of adipocytes, contribute to the formation of important structural elements, including collagen, elastin, and ECM proteins [36]. In our study, the Masson staining results suggested that an abnormal macrophage polarization results in deficient ECM formation. All of the aforementioned findings support the notion that reprogramming macrophage polarization could be a new therapeutic strategy for treating diabetic nonhealing wounds.

Recent studies have demonstrated that Exos can transfer mature miRs between organs, resulting in functional changes in the receiving organs and the alleviation of T2DM [37]. Emerging evidence suggests that the states of donor cell determine the function of Exos via the transport of miRs into neighboring or distant cells [38]. Previous research revealed that lean mice produce adipose tissue Exos that directly enhance insulin signaling in vitro and, when administered to obese mice, markedly improve insulin sensitivity and glucose tolerance, two important factors that can lead to macrophage polarization equilibrium [39, 40]. Importantly, we report that Exos^Lean^-treated mice exhibit balanced macrophage polarization and enhanced wound healing. This effect, however, may be due to the Exos injection-induced modulation of inflammation. To avoid this unintended effect, control Exos were also administered. Compared with Exos^Lean^, it was discovered that control Exos did not exert a significant therapeutic effect. Exos ex vivo enables the release of lipids from the adipose tissue from lean mice, and the rate of lipid release in obese animals more than doubles. In vitro differentiation of bone marrow precursors into ATM-like cells can be induced by these adipose Exos and their associated factors [41]. Given that macrophages in lean adipose tissue are predominantly anti-inflammatory M2-like cells [37], lean ATMs may facilitate the beneficial transition of ATMs through a positive feedback mechanism. The reprogramming of macrophage polarization by Exos^Lean^ was also observed in our study.

To further explore which exosomal miRs or miRs groups contribute to immunomodulatory phenotypes, we sequenced miRs from db/db mice and Exos^Lean^-treated db/db mice. We found that, among the miRs highly enriched in Exos^Lean^-treated mice, miR-222-3p serves as a novel effector since its beneficial effects on metabolism are involved in tissue regeneration [42]. MiR-222, which is enriched in the culture medium of adipose tissue stem cells, modulates inflammation and protects cells from apoptosis, has been the focus of research [43]. Our results revealed that ATM-Exos isolated from adipose tissue-conditioned medium not only led to miR-222-3p accumulation in macrophages, but also induced the transformation of M1 macrophages into M2 macrophages by activating a transcriptional program that is characteristic of the M2 phenotype. Overexpression of miR-222 has been shown to inhibit the expression of several inflammatory mediators, such as TNF-α, at the protein, mRNA, and transcript levels. Conversely, inhibition of miR-222 decreased anti-inflammatory gene expression, even during a naïve LPS response [44], which is consistent with our in vitro finding that treatment with a miR-222-3p inhibitor decreased M2 polarization.

To further elucidate the mechanisms underlying the effects of miR-222-3p on macrophage polarization, translational arrest and mRNA degradation of miRs-bound mRNAs were identified. Our analysis revealed that miR-222-3p repressed the expression of a series of mRNAs associated with processes such as immune modulation and repression of cellular apoptosis [30, 45]. Among the mRNAs repressed by miR-222-3p, Bcl2l11/Bim, the pro-apoptotic BH3-only gene that leads to cellular apoptosis and promotes an inflammatory response, was chosen for further study [46]. Bim acts as a crucial regulator of apoptosis, and this extrinsic cell death pathway was also initially believed to control the immune response by regulating the availability of type 1 cytokines, such as IL-15, as previously reported [47]. Further, type 1 immune response restrained macrophage polarization [48]. In addition, we found that Bim is the authentic mRNA target of miR-222-3p. The expression of Bim increased following the inhibition of miR-222-3p in macrophages, demonstrating the inhibitory effects of miR-222-3p on Bim. Bim has been identified as a key mediator of both pancreatic β-cell death and hepatic insulin resistance and is thus a potential therapeutic target for diabetes [49]. Together with previous studies, our data indicate that Exos^Lean^, as an effective diabetes protector, induced miR-222-3p activation, leading to Bim inhibition, thereby affecting diabetes wound healing.

In summary, chronic diabetic wounds are accompanied by an accumulation of pro-inflammatory macrophage, which leads to deficient wound healing. In addition, we found that lean adipose tissue secretes miRs-loaded Exos that affect macrophage polarization. Treatment with Exos^Lean^ induces M2 polarization in vivo and in vitro, thereby improving wound healing. Exos^Lean^ treatment increases the expression of miR-222-3p and reduces Bim expression. The resulting changes in Bim expression led to re-polarization of macrophage. This study advances our understanding of chronic diabetic wound healing and identifies novel therapeutic targets that can be exploited to design effective interventions that enhance wound healing in diabetes patients.

## Supplementary Information

**Additional file 1: Fig. S1.** Regulation of exosomal miR-222-3p on expression of Bim in vivo. Western blotting was used to analyze Bim and β-actin protein levels in db/db mic treated with PBS, Exos^Lean^, Exos^Lean^ + miR-222-3p inhibitor, or Exos^Lean^ + miR-NC inhibitor. (A) Typical pictures of each group blots; (B) Statistical analysis of ratio of Bim to β-actin; ^*^P < 0.05, ^#^*P* < 0.05 by one-way analysis of variance (n = 3).

**Additional file 2: Fig. S2.** Depression of Bim induced by Exos^Lean^ confers effects on wound healing and immune-regulation. (A) Representative images of wound closure in the different treatment groups. (B) The relative wound area was calculated; (C and D) Immunofluorescence staining image showing the localization of CD68+/CD86+ M1 macrophages and statistical analysis of the proportion of M1 macrophages; (E and F) Immunofluorescence staining image showing the localization of CD68+/CD206+ M2 macrophages and statistical analysis of the proportion of M2 macrophages; G1: Exos^Lean^; G2: Exos^Lean^ + AAV9-Bim1; ^*^P < 0.05 by one-way analysis of variance (n = 3).

## Data Availability

All data and materials are available in the manuscript.

## Abbreviations

miRs: MicroRNAs
Exos: Exosomes
ATMs: Adipose tissue macrophages
ATM-Exos: Exosomes secreted by ATMs
Exos^Lean^: Exosomes secreted by lean ATMs
NCDs: Priority noncommunicable diseases
T2DM: Type 2 diabetes mellitus
Bcl2l11: Bcl-2-like protein 11
db/db: B6.BKS(D)-Leprdb/J
VATs: Visceral adipose tissues
TEM: Transmission electron microscopy
NTA: Nanoparticle tracking analysis
qRT-PCR: Quantitative real-time RT-PCR
iNOS: Inducible nitric oxide synthase
TNF-ɑ: Tumor necrosis factor-ɑ
IL-1β: Interleukin-1β
Arg1: Arginine1
TGF-β1: Transforming growth factor-β1
IL-10: Interleukin-10
CD31: Platelet endothelial cell adhesion molecule-1

## References

[1] Duncan BB, Schmidt MI (2022). Diabetes mortality and trends before 25 years of age: an analysis of the Global Burden of Disease Study 2019. Lancet Diabetes Endocrinol.

[2] Moucheraud C, Lenz C, Latkovic M, Wirtz VJ (2019). The costs of diabetes treatment in low- and middle-income countries: a systematic review. BMJ Glob Health.

[3] Brem H, Tomic-Canic M (2007). Cellular and molecular basis of wound healing in diabetes. J Clin Invest.

[4] Sancar G, Liu S, Gasser E, Alvarez JG, Moutos C, Kim K, van Zutphen T, Wang Y, Huddy TF, Ross B (2022). FGF1 and insulin control lipolysis by convergent pathways. Cell Metab.

[5] Kong M, Xie K, Lv M, Li J, Yao J, Yan K, Wu X, Xu Y, Ye D (2021). Anti-inflammatory phytochemicals for the treatment of diabetes and its complications: lessons learned and future promise. Biomed Pharmacother.

[6] Morton LM, Phillips TJ (2016). Wound healing and treating wounds: differential diagnosis and evaluation of chronic wounds. J Am Acad Dermatol.

[7] Wynn TA, Vannella KM (2016). Macrophages in tissue repair, regeneration, and fibrosis. Immunity.

[8] Yan J, Tie G, Wang S, Tutto A, DeMarco N, Khair L, Fazzio TG, Messina LM (2018). Diabetes impairs wound healing by Dnmt1-dependent dysregulation of hematopoietic stem cells differentiation towards macrophages. Nat Commun.

[9] Qian Y, Zheng Y, Jin J, Wu X, Xu K, Dai M, Niu Q, Zheng H, He X, Shen J (2022). Immunoregulation in diabetic wound repair with a photoenhanced glycyrrhizic acid hydrogel scaffold. Adv Mater.

[10] Crewe C, Funcke JB, Li S, Joffin N, Gliniak CM, Ghaben AL, An YA, Sadek HA, Gordillo R, Akgul Y (2021). Extracellular vesicle-based interorgan transport of mitochondria from energetically stressed adipocytes. Cell Metab.

[11] Crewe C, An YA, Scherer PE (2017). The ominous triad of adipose tissue dysfunction: inflammation, fibrosis, and impaired angiogenesis. J Clin Invest.

[12] Rohm TV, Meier DT, Olefsky JM, Donath MY (2022). Inflammation in obesity, diabetes, and related disorders. Immunity.

[13] Ying W, Riopel M, Bandyopadhyay G, Dong Y, Birmingham A, Seo JB, Ofrecio JM, Wollam J, Hernandez-Carretero A, Fu W (2017). Adipose tissue macrophage-derived exosomal miRNAs can modulate in vivo and in vitro insulin sensitivity. Cell.

[14] Shan B, Wang X, Wu Y, Xu C, Xia Z, Dai J, Shao M, Zhao F, He S, Yang L (2017). The metabolic ER stress sensor IRE1α suppresses alternative activation of macrophages and impairs energy expenditure in obesity. Nat Immunol.

[15] Russo L, Lumeng CN (2018). Properties and functions of adipose tissue macrophages in obesity. Immunology.

[16] Girón-Ulloa A, González-Domínguez E, Klimek RS, Patiño-Martínez E, Vargas-Ayala G, Segovia-Gamboa NC, Campos-Peña V, Rodríguez-Arellano ME, Meraz-Ríos MA, Campos-Campos SF, Sánchez-Torres C (2020). Specific macrophage subsets accumulate in human subcutaneous and omental fat depots during obesity. Immunol Cell Biol.

[17] Gesmundo I, Pardini B, Gargantini E, Gamba G, Birolo G, Fanciulli A, Banfi D, Congiusta N, Favaro E, Deregibus MC (2021). Adipocyte-derived extracellular vesicles regulate survival and function of pancreatic β cells. JCI Insight.

[18] Zhao H, Shang Q, Pan Z, Bai Y, Li Z, Zhang H, Zhang Q, Guo C, Zhang L, Wang Q (2018). Exosomes from adipose-derived stem cells attenuate adipose inflammation and obesity through polarizing M2 macrophages and Beiging in white adipose tissue. Diabetes.

[19] Huang-Doran I, Zhang CY, Vidal-Puig A (2017). Extracellular vesicles: novel mediators of cell communication in metabolic disease. Trends Endocrinol Metab.

[20] Fong MY, Zhou W, Liu L, Alontaga AY, Chandra M, Ashby J, Chow A, O'Connor ST, Li S, Chin AR (2015). Breast-cancer-secreted miR-122 reprograms glucose metabolism in premetastatic niche to promote metastasis. Nat Cell Biol.

[21] Kahn CR, Wang G, Lee KY (2019). Altered adipose tissue and adipocyte function in the pathogenesis of metabolic syndrome. J Clin Invest.

[22] Arner P, Kulyté A (2015). MicroRNA regulatory networks in human adipose tissue and obesity. Nat Rev Endocrinol.

[23] Boutens L, Hooiveld GJ, Dhingra S, Cramer RA, Netea MG, Stienstra R (2018). Unique metabolic activation of adipose tissue macrophages in obesity promotes inflammatory responses. Diabetologia.

[24] Wu J, Li X, Li D, Ren X, Li Y, Herter EK, Qian M, Toma MA, Wintler AM, Sérézal IG (2020). MicroRNA-34 family enhances wound inflammation by targeting LGR4. J Invest Dermatol.

[25] Ayesha M, Majid A, Zhao D, Greenaway FT, Yan N, Liu Q, Liu S, Sun MZ (2022). MiR-4521 plays a tumor repressive role in growth and metastasis of hepatocarcinoma cells by suppressing phosphorylation of FAK/AKT pathway via targeting FAM129A. J Adv Res.

[26] Thangaraj A, Chivero ET, Tripathi A, Singh S, Niu F, Guo ML, Pillai P, Periyasamy P, Buch S (2021). HIV TAT-mediated microglial senescence: role of SIRT3-dependent mitochondrial oxidative stress. Redox Biol.

[27] Sharifiaghdam M, Shaabani E, Sharifiaghdam Z, De Keersmaecker H, Lucas B, Lammens J, Ghanbari H, Teimoori-Toolabi L, Vervaet C, De Beer T (2021). Macrophage reprogramming into a pro-healing phenotype by siRNA delivered with LBL assembled nanocomplexes for wound healing applications. Nanoscale.

[28] Zhao G, Hochwalt PC, Usui ML, Underwood RA, Singh PK, James GA, Stewart PS, Fleckman P, Olerud JE (2010). Delayed wound healing in diabetic (db/db) mice with *Pseudomonas aeruginosa* biofilm challenge: a model for the study of chronic wounds. Wound Repair Regen.

[29] Boniakowski AE, Kimball AS, Jacobs BN, Kunkel SL, Gallagher KA (2017). Macrophage-mediated inflammation in normal and diabetic wound healing. J Immunol.

[30] Corsten MF, Heggermont W, Papageorgiou AP, Deckx S, Tijsma A, Verhesen W, van Leeuwen R, Carai P, Thibaut HJ, Custers K (2015). The microRNA-221/-222 cluster balances the antiviral and inflammatory response in viral myocarditis. Eur Heart J.

[31] Wong SL, Demers M, Martinod K, Gallant M, Wang Y, Goldfine AB, Kahn CR, Wagner DD (2015). Diabetes primes neutrophils to undergo NETosis, which impairs wound healing. Nat Med.

[32] Shook BA, Wasko RR, Mano O, Rutenberg-Schoenberg M, Rudolph MC, Zirak B, Rivera-Gonzalez GC, López-Giráldez F, Zarini S, Rezza A (2020). Dermal adipocyte lipolysis and myofibroblast conversion are required for efficient skin repair. Cell Stem Cell.

[33] Louiselle AE, Niemiec SM, Zgheib C, Liechty KW (2021). Macrophage polarization and diabetic wound healing. Transl Res.

[34] Kimball AS, Davis FM, denDekker A, Joshi AD, Schaller MA, Bermick J, Xing X, Burant CF, Obi AT, Nysz D (2019). The Histone methyltransferase Setdb2 modulates macrophage phenotype and uric acid production in diabetic wound repair. Immunity.

[35] Chazaud B (2020). Inflammation and skeletal muscle regeneration: leave it to the macrophages!. Trends Immunol.

[36] Shook BA, Wasko RR, Rivera-Gonzalez GC, Salazar-Gatzimas E, López-Giráldez F, Dash BC, Muñoz-Rojas AR, Aultman KD, Zwick RK, Lei V (2018). Myofibroblast proliferation and heterogeneity are supported by macrophages during skin repair. Science.

[37] Ying W, Gao H, Dos Reis FCG, Bandyopadhyay G, Ofrecio JM, Luo Z, Ji Y, Jin Z, Ly C, Olefsky JM (2021). MiR-690, an exosomal-derived miRNA from M2-polarized macrophages, improves insulin sensitivity in obese mice. Cell Metab.

[38] Castaño C, Kalko S, Novials A, Párrizas M (2018). Obesity-associated exosomal miRNAs modulate glucose and lipid metabolism in mice. Proc Natl Acad Sci U S A.

[39] Barboza E, Hudson J, Chang WP, Kovats S, Towner RA, Silasi-Mansat R, Lupu F, Kent C, Griffin TM (2017). Profibrotic infrapatellar fat pad remodeling without M1 macrophage polarization precedes knee osteoarthritis in mice with diet-induced obesity. Arthritis Rheumatol.

[40] Haschemi A, Kosma P, Gille L, Evans CR, Burant CF, Starkl P, Knapp B, Haas R, Schmid JA, Jandl C (2012). The sedoheptulose kinase CARKL directs macrophage polarization through control of glucose metabolism. Cell Metab.

[41] Flaherty SE, Grijalva A, Xu X, Ables E, Nomani A, Ferrante AW (2019). A lipase-independent pathway of lipid release and immune modulation by adipocytes. Science.

[42] Liu X, Xiao J, Zhu H, Wei X, Platt C, Damilano F, Xiao C, Bezzerides V, Boström P, Che L (2015). miR-222 is necessary for exercise-induced cardiac growth and protects against pathological cardiac remodeling. Cell Metab.

[43] Lee TL, Lai TC, Lin SR, Lin SW, Chen YC, Pu CM, Lee IT, Tsai JS, Lee CW, Chen YL (2021). Conditioned medium from adipose-derived stem cells attenuates ischemia/reperfusion-induced cardiac injury through the microRNA-221/222/PUMA/ETS-1 pathway. Theranostics.

[44] Seeley JJ, Baker RG, Mohamed G, Bruns T, Hayden MS, Deshmukh SD, Freedberg DE, Ghosh S (2018). Induction of innate immune memory via microRNA targeting of chromatin remodelling factors. Nature.

[45] Li L, Sheng P, Li T, Fields CJ, Hiers NM, Wang Y, Li J, Guardia CM, Licht JD, Xie M (2021). Widespread microRNA degradation elements in target mRNAs can assist the encoded proteins. Genes Dev.

[46] Iurlaro R, Muñoz-Pinedo C (2016). Cell death induced by endoplasmic reticulum stress. Febs J.

[47] Kurtulus S, Sholl A, Toe J, Tripathi P, Raynor J, Li KP, Pellegrini M, Hildeman DA (2015). Bim controls IL-15 availability and limits engagement of multiple BH3-only proteins. Cell Death Differ.

[48] Liu G, Zhai Q, Schaffner D, Bradburne C, Wu A, Hayford A, Popov S, Grene E, Bailey C, Alibek K (2004). IL-15 induces IFN-beta and iNOS gene expression, and antiviral activity of murine macrophage RAW 264.7 cells. Immunol Lett.

[49] Miani M, Elvira B, Gurzov EN (2018). Sweet killing in obesity and diabetes: the metabolic role of the BH3-only protein BIM. J Mol Biol.

