# Supplementary figures and images for: Lean adipose tissue macrophage derived exosome confers immunoregulation to improve wound healing in diabetes
Source: J Nanobiotechnology. 2023 Apr 12;21:128. doi: 10.1186/s12951-023-01869-4 (PMC10091677; doi:10.1186/s12951-023-01869-4)

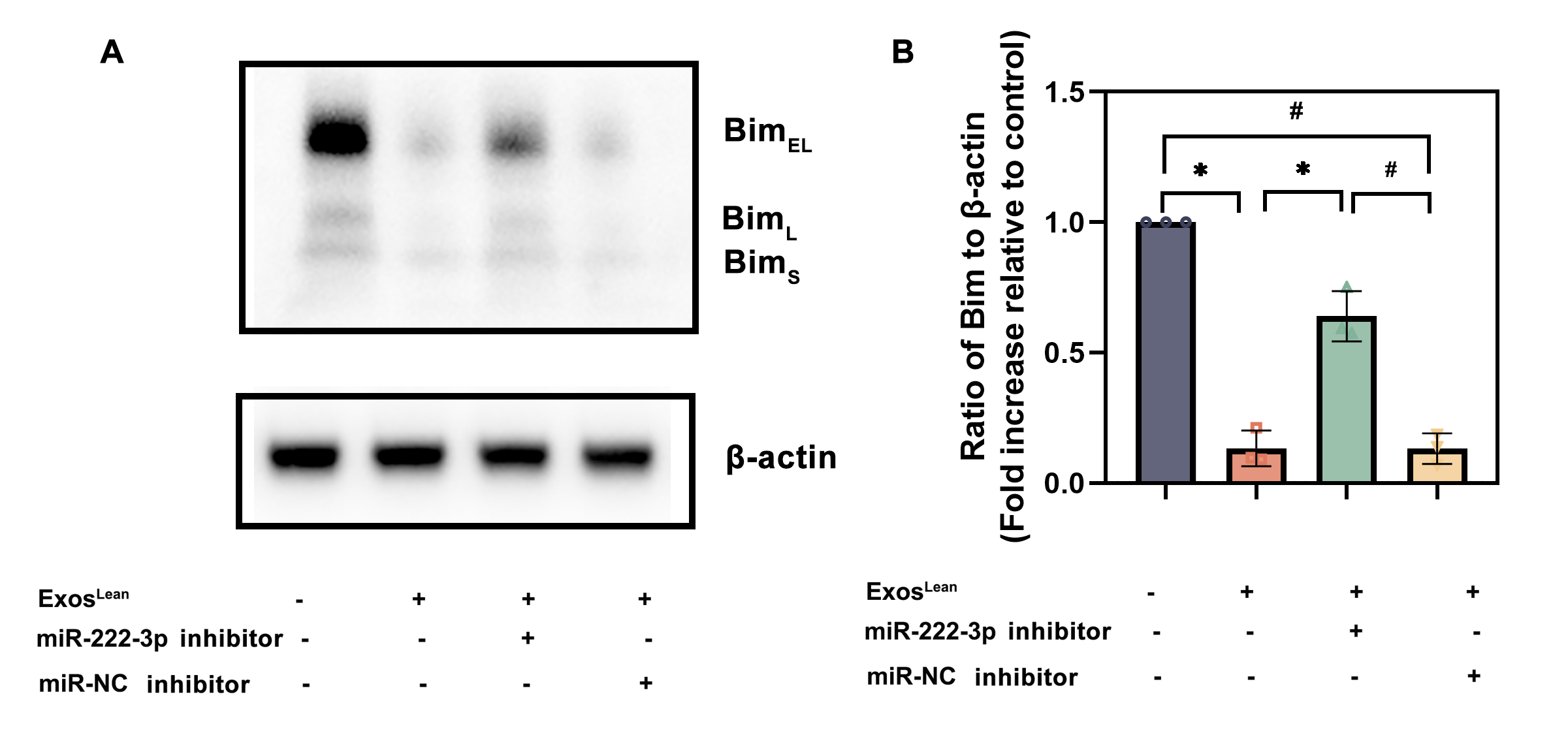

Supplement: Supplementary file 1 — Additional file 1: Fig. S1. Regulation of exosomal miR-222-3p on expression of Bim in vivo. Western blotting was used to analyze Bim and β-actin protein levels in db/db mic treated with PBS, ExosLean, ExosLean + miR-222-3p inhibitor, or ExosLean + miR-NC inhibitor. (A) Typical pictures of each group blots; (B) Statistical analysis of ratio of Bim to β-actin; *P < 0.05, #P < 0.05 by one-way analysis of variance (n = 3). [file 12951_2023_1869_MOESM1_ESM.tif]

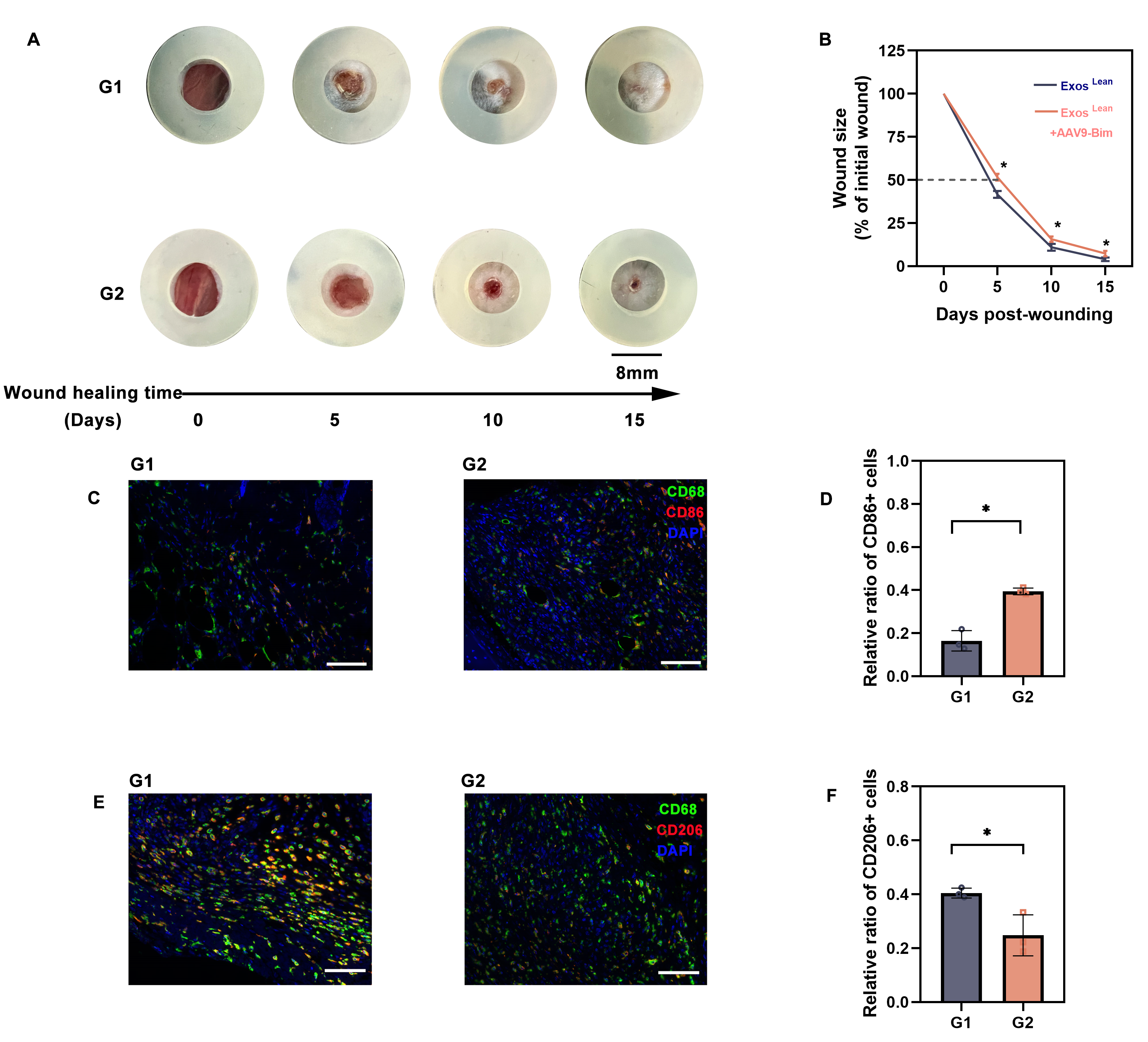

Supplement: Supplementary file 2 — Additional file 2: Fig. S2. Depression of Bim induced by ExosLean confers effects on wound healing and immune-regulation. (A) Representative images of wound closure in the different treatment groups. (B) The relative wound area was calculated; (C and D) Immunofluorescence staining image showing the localization of CD68+/CD86+ M1 macrophages and statistical analysis of the proportion of M1 macrophages; (E and F) Immunofluorescence staining image showing the localization of CD68+/CD206+ M2 macrophages and statistical analysis of the proportion of M2 macrophages; G1: ExosLean; G2: ExosLean + AAV9-Bim1; *P < 0.05 by one-way analysis of variance (n = 3). [file 12951_2023_1869_MOESM2_ESM.tif]
